# An Unusual Case of Tuberculous Tenosynovitis of the Hand

**DOI:** 10.7759/cureus.63081

**Published:** 2024-06-24

**Authors:** Manimaran R, Koppolu Kanchana, Aiswerya Shankar, Karthick J

**Affiliations:** 1 Plastic Surgery, Sree Balaji Medical College & Hospital, Chennai, IND; 2 General Surgery, Sree Balaji Medical College & Hospital, Chennai, IND; 3 Plastic and Reconstructive Surgery, Sree Balaji Medical College & Hospital, Chennai, IND

**Keywords:** tuberculous tenosynovitis, multinucleated giant cells, rice bodies, mycobacterium tuberculosis, tenosynovitis of wrist

## Abstract

Infectious tuberculous tenosynovitis (TS) is an unusual occurrence in the forearm, wrist, or hand. Here we report a case of tuberculous TS of the wrist in a 26-year-old male with no comorbidities. The patient presented with a nonhealing ulcer on the palmar aspect of the proximal part of the left little finger with restricted mobility. There were no other symptoms to confirm the existence of an active tuberculosis infection in this patient. This case report helps broaden our knowledge about the different presentations of tuberculous TS in a patient with no history of exposure to tuberculosis.

## Introduction

Chronic tuberculous tenosynovitis (CTT) is a rare condition affecting the wrist and the hand, especially in endemic areas. It accounts for 5% of extrapulmonary tuberculous cases involving the musculotendinous system and skeletal system [1]. Tenosynovitis (TS) of the upper limb could be infectious, noninfectious, or idiopathic in etiology. Tuberculous TS of the upper limbs is more common in the wrist, especially the ulnar bursa [1,2]. Of the infective etiology, the most common causative organism of TS is *Staphylococcus aureus*, which affects the tendons of flexion in the wrist [3]. Although the clinical presentation of chronic TS due to tuberculous etiology is not specific, patients generally present with a swelling in the hand and a history of long-standing dull aching pain. Due to these nonspecific complaints and delayed presentation, this condition is difficult to diagnose and treat [4].

Histopathological examination is the gold standard for confirmation of the diagnosis, and patients are generally treated with antituberculosis therapy (ATT). Surgical management in CTT is indicated in patients with acute secondary infections, nerve compression, or failure of conservative management. Here we present the case of a 26-year-old male patient with CTT who presented with nerve compression and underwent surgical management.

## Case presentation

**Figure 1:**
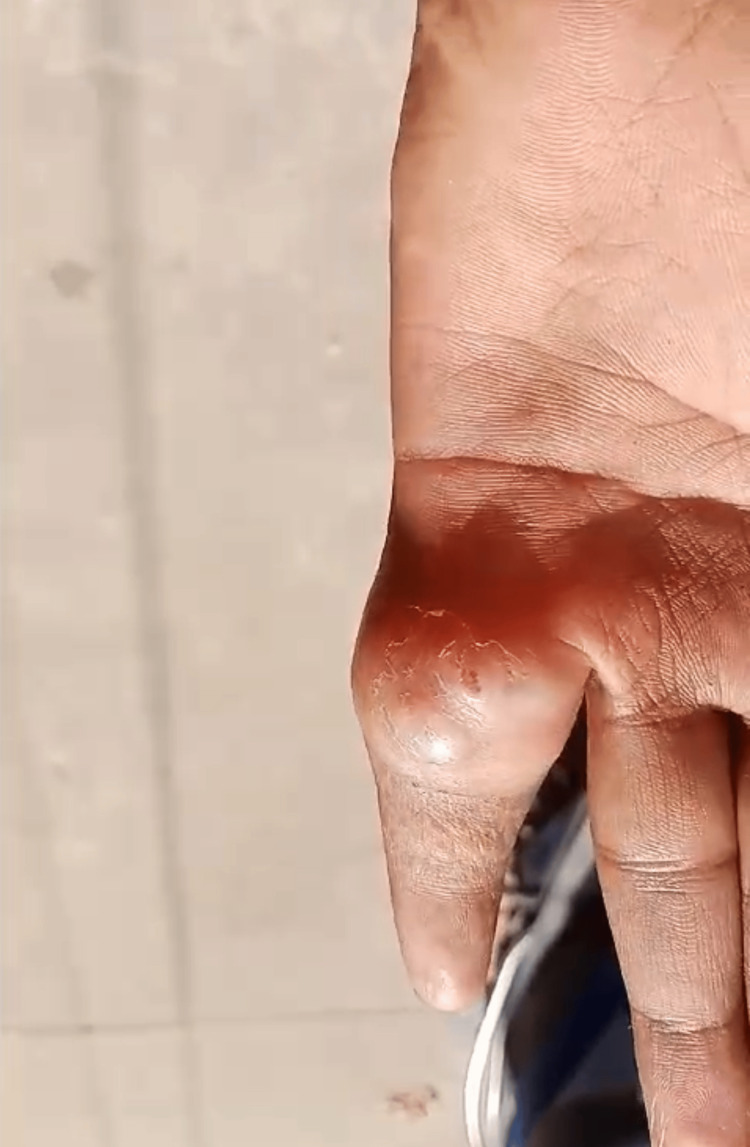
Swelling at the base of the left little finger

**Figure 2:**
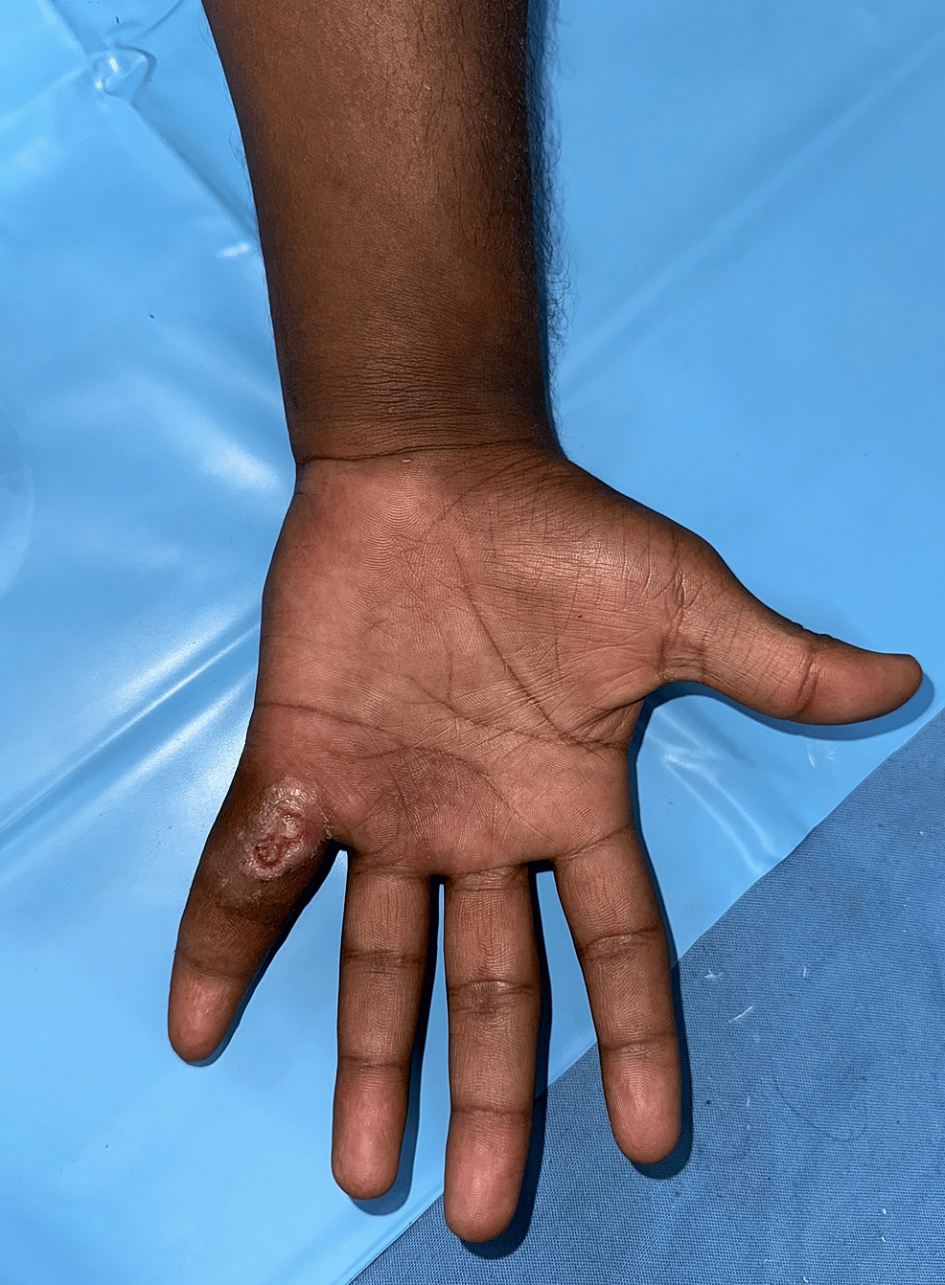
Gradual progression of swelling into a nonhealing ulcer

A 26-year-old male presented to the Plastic Surgery outpatient department at Sree Balaji Medical College & Hospital, Chennai, India, with a nonhealing ulcer at the base of the left little finger. He was a student who had suffered trauma while lifting a water can. Although the patient did not provide a clear history of trauma, a swelling developed at the base of his little finger two days after the incident. The swelling, initially painless and measuring 2 × 2.5 cm (Figure 1), gradually increased in size. Eventually, it spontaneously burst, releasing serous discharge and leading to the formation of an ulcer in September 2023. The patient sought care at the local primary health care center twice, where he received a course of antibiotics. Despite this treatment, the ulcer persisted without reduction in size. In November 2023, he presented to our department with a nonhealing ulcer (Figure 2) that showed no further enlargement and had no active discharge. He reported experiencing dull, aching pain and discomfort, which limited the range of motion of his left little finger. He denied experiencing paresthesia, numbness, or tingling sensations. The patient did not report symptoms such as an evening rise in temperature, night sweats, breathlessness, or a chronic cough. He also had no history of tuberculosis contact.

**Figure 3:**
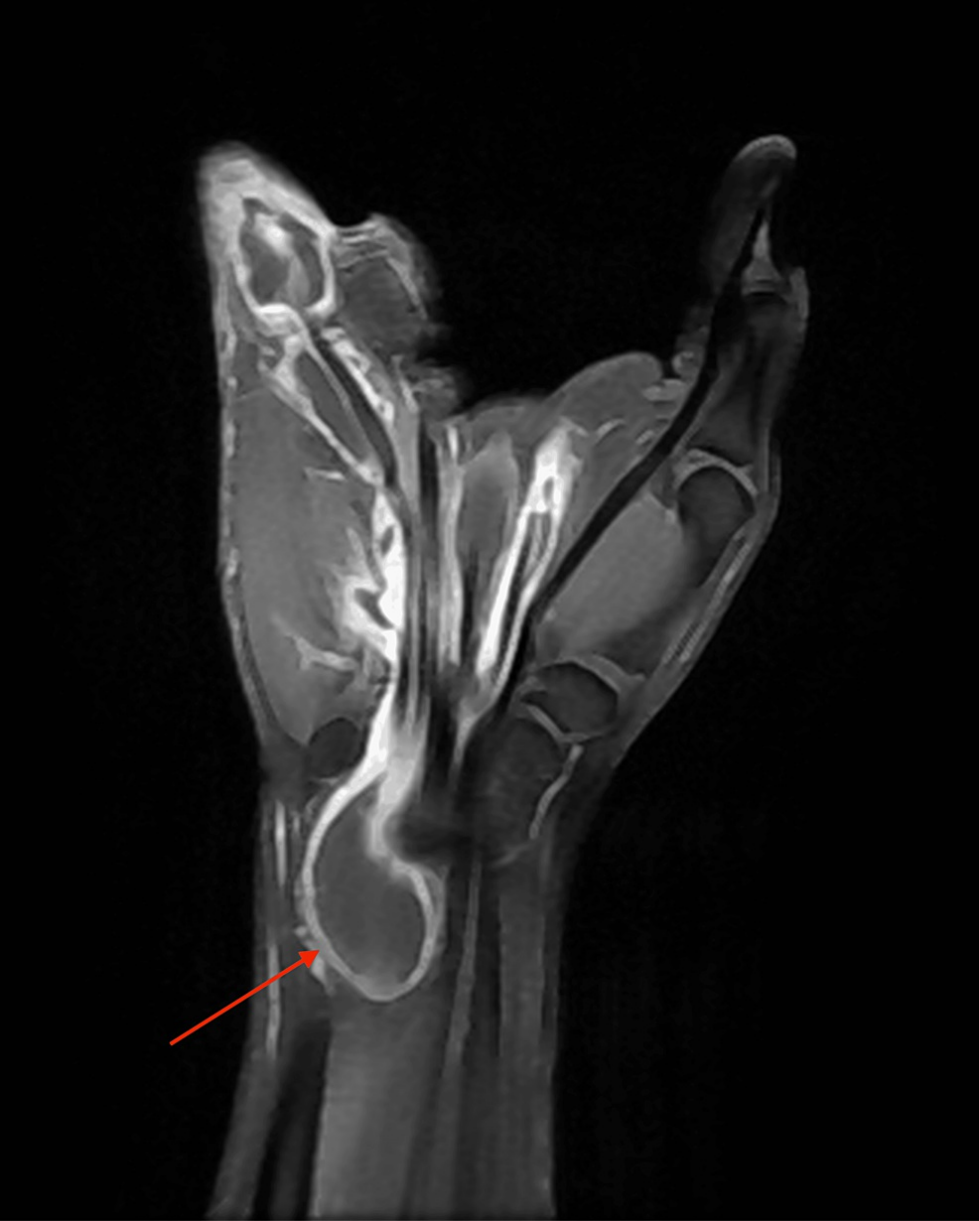
MRI of the left hand in coronal view showing diffuse thickening and enhancement of the tendon sheath of the flexor digitorum superficialis

**Figure 4:**
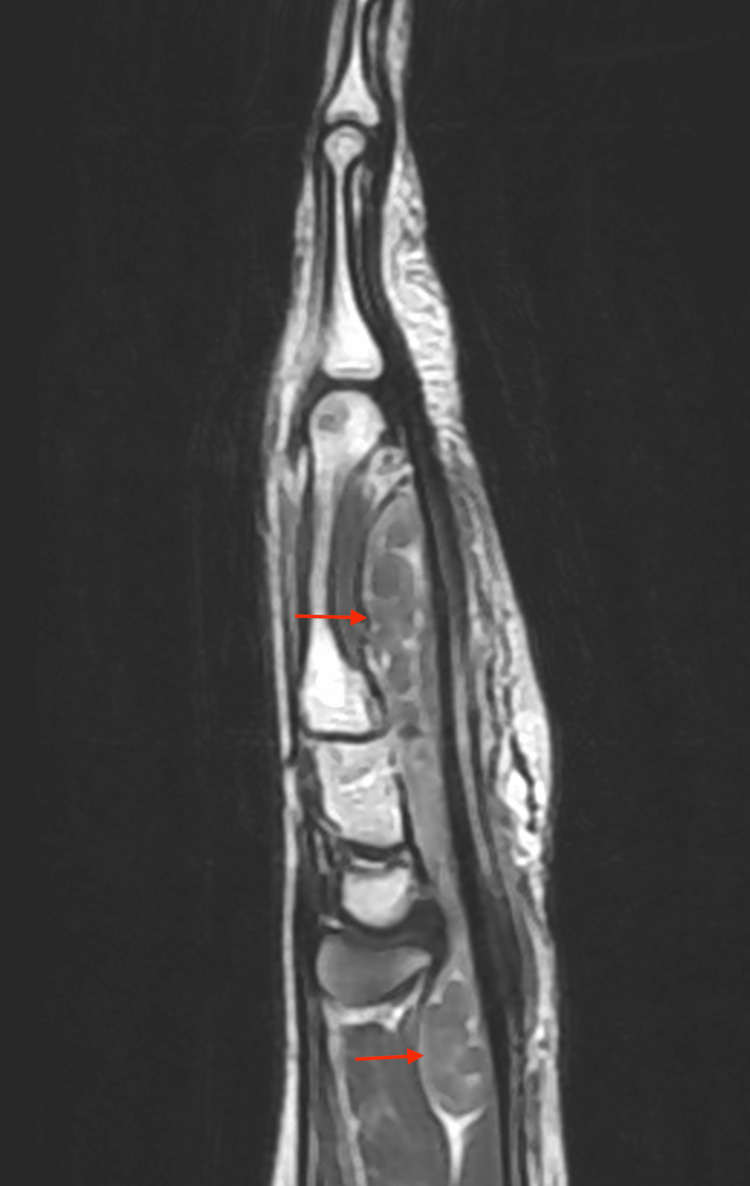
MRI of the left hand in sagittal view showing diffuse thickening and non-enhancing nodules in the tendon sheath of the flexor digitorum profundus and flexor digitorum superficialis

**Figure 5:**
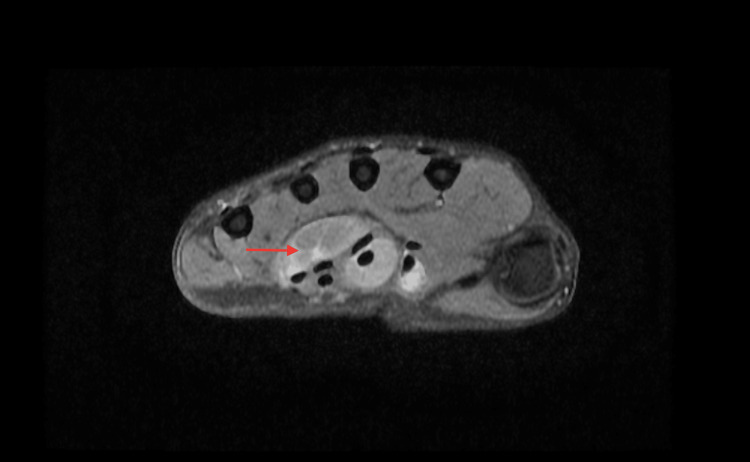
MRI of the left hand in axial view showing diffuse thickening of the synovium and non-enhancing nodules in the tendon sheath of the flexor digitorum superficialis

His general examination revealed normal vitals with no evidence of pallor, icterus, or generalized lymphadenopathy. He appeared moderately built and well nourished. On local examination, a well-defined ulcer measuring 1 × 0.5 cm was noted on the palmar aspect of the base of his left little finger, covered with slough and without active discharge. Edema was observed at the base of the left little finger, accompanied by a 2 × 2.5 cm globular swelling below the hypothenar eminence of the left wrist. The swelling was soft in consistency, with minimal fluctuation and limited mobility. The clinical assessment indicated restricted movement at the metacarpophalangeal joint, proximal, and distal interphalangeal joints of the left little finger, as well as at the wrist during flexion. All other fingers demonstrated a normal range of motion, and there was no axillary or cervical lymphadenopathy. Laboratory investigations revealed a leukocyte count of 7.7 × 10^9^/L, an erythrocyte sedimentation rate (ESR) of 10 mm/hr, and a hemoglobin level of 14.2 g/dL. The Mantoux test was negative. A chest X-ray showed no parenchymal lesions, and an X-ray of the left hand revealed no bony abnormalities or signs of osteomyelitis. MRI findings of the left hand demonstrated diffuse synovial thickening and enhancement along the flexor digitorum superficialis and flexor digitorum profundus tendons in the distal forearm, carpal tunnel, palm of the hand, and little finger. Multiple non-enhancing nodular lesions of mildly T2 intermediate signal intensity, measuring 5-13 mm, were observed distending the synovium of the affected tendons (Figures 3, 4, 5). Additionally, a soft tissue defect was identified on the volar aspect of the left finger at the proximal phalanx level. Compression of the median nerve within the carpal tunnel due to diseased flexor tendons was noted, while other carpal bones, metacarpals, phalanges, and extensor tendons appeared normal.

**Figure 6:**
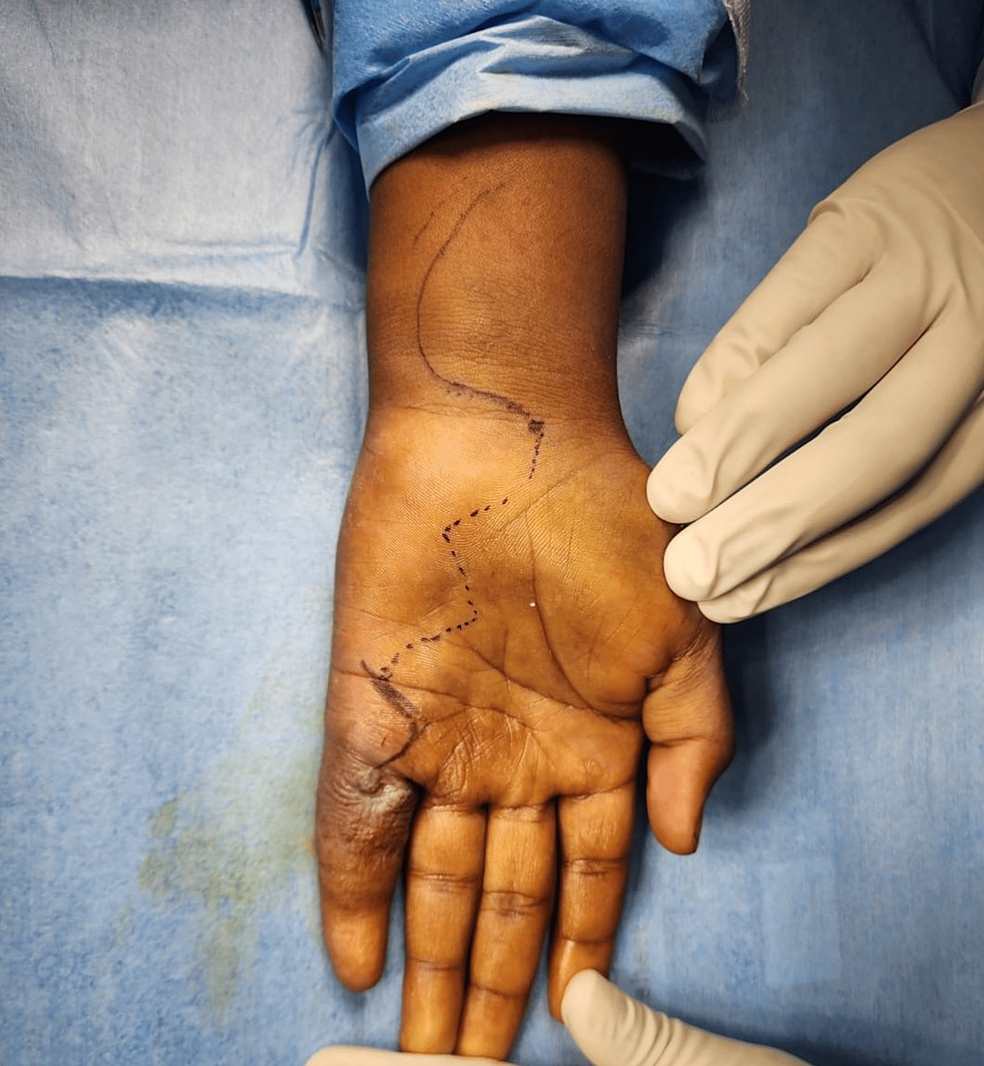
Preoperative marking of the zigzag incision using a sterile marker

**Figure 7:**
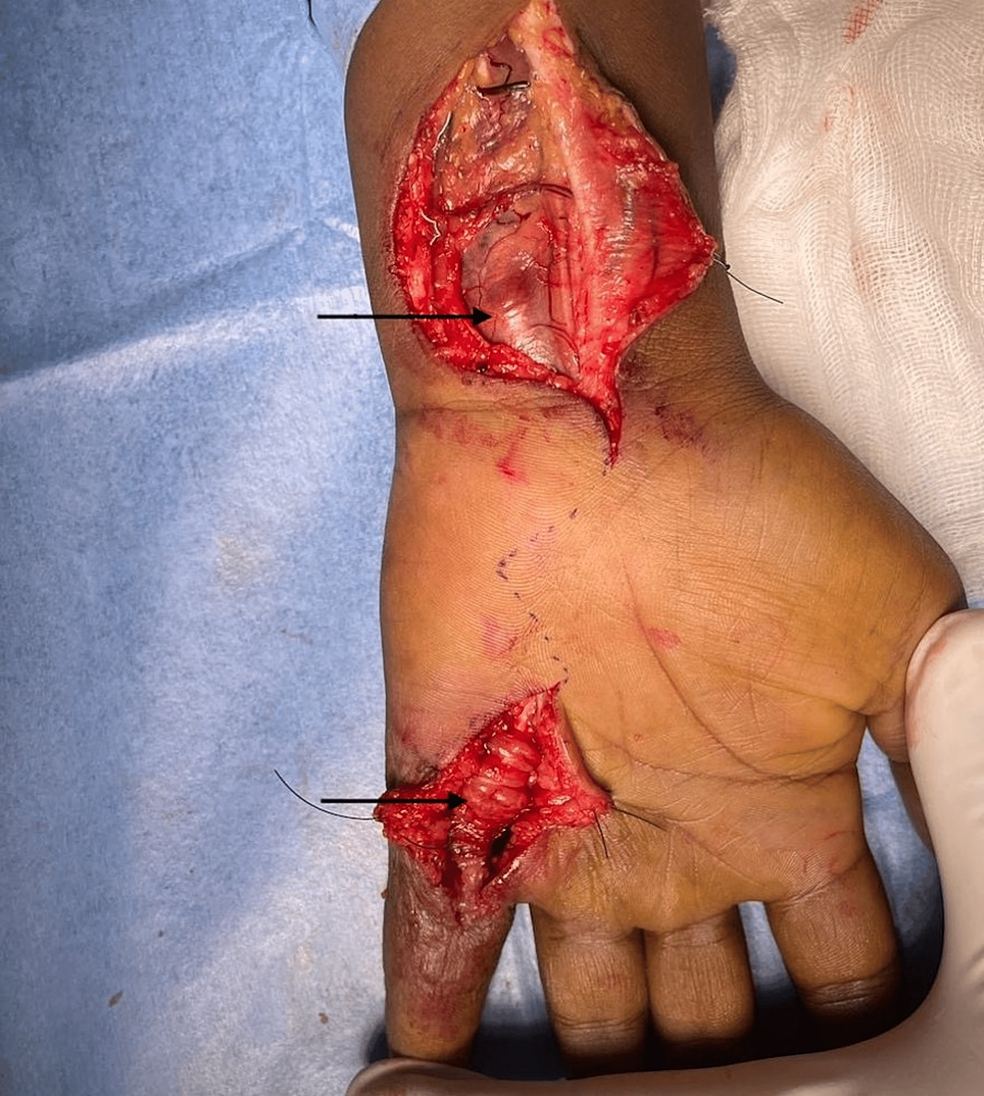
Intraoperative image showing multiple marked swellings surrounding the tendons

**Figure 8:**
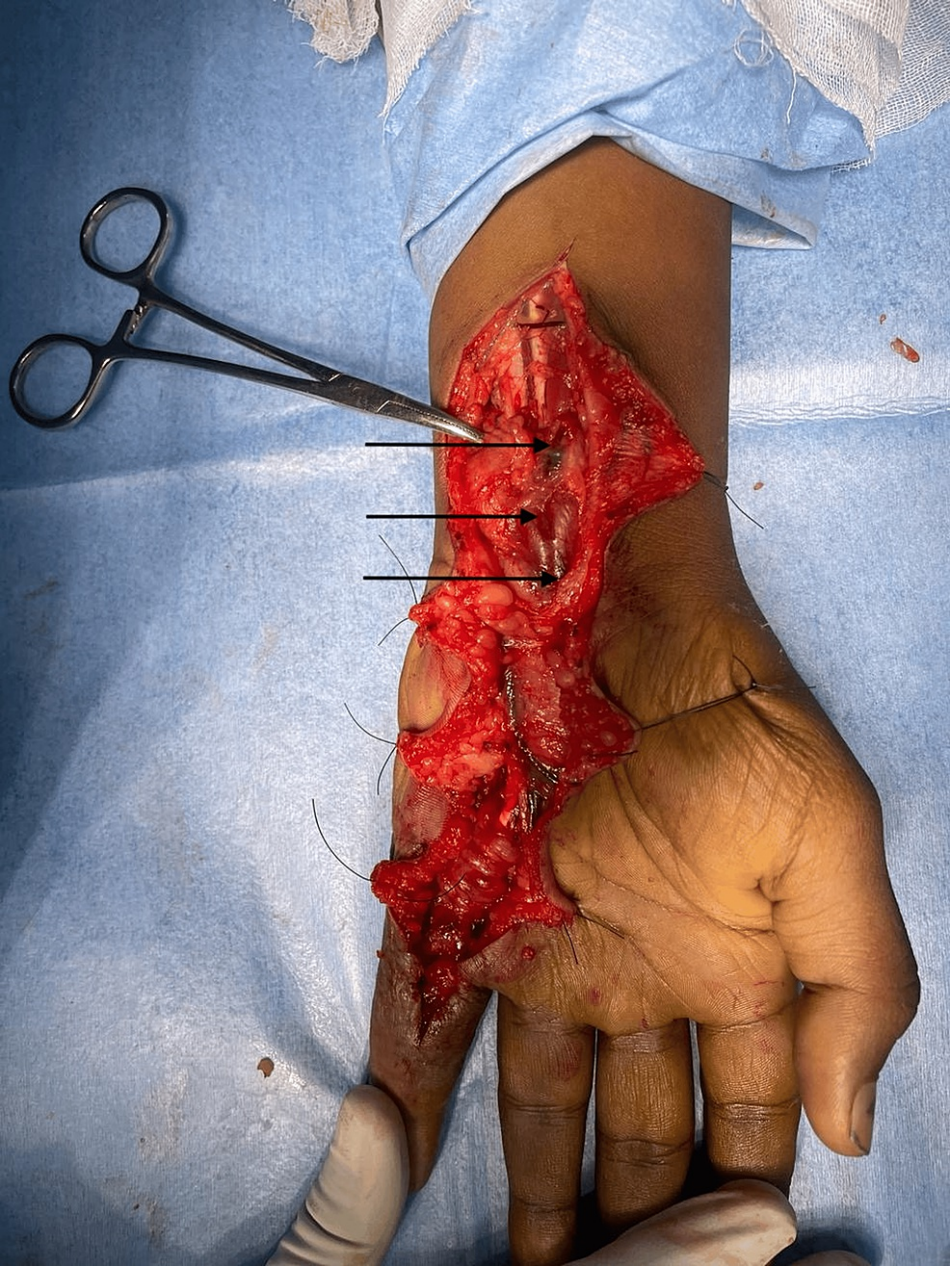
Intraoperative image showing several swellings within the carpal tunnel, potentially causing compression of the median nerve

**Figure 9:**
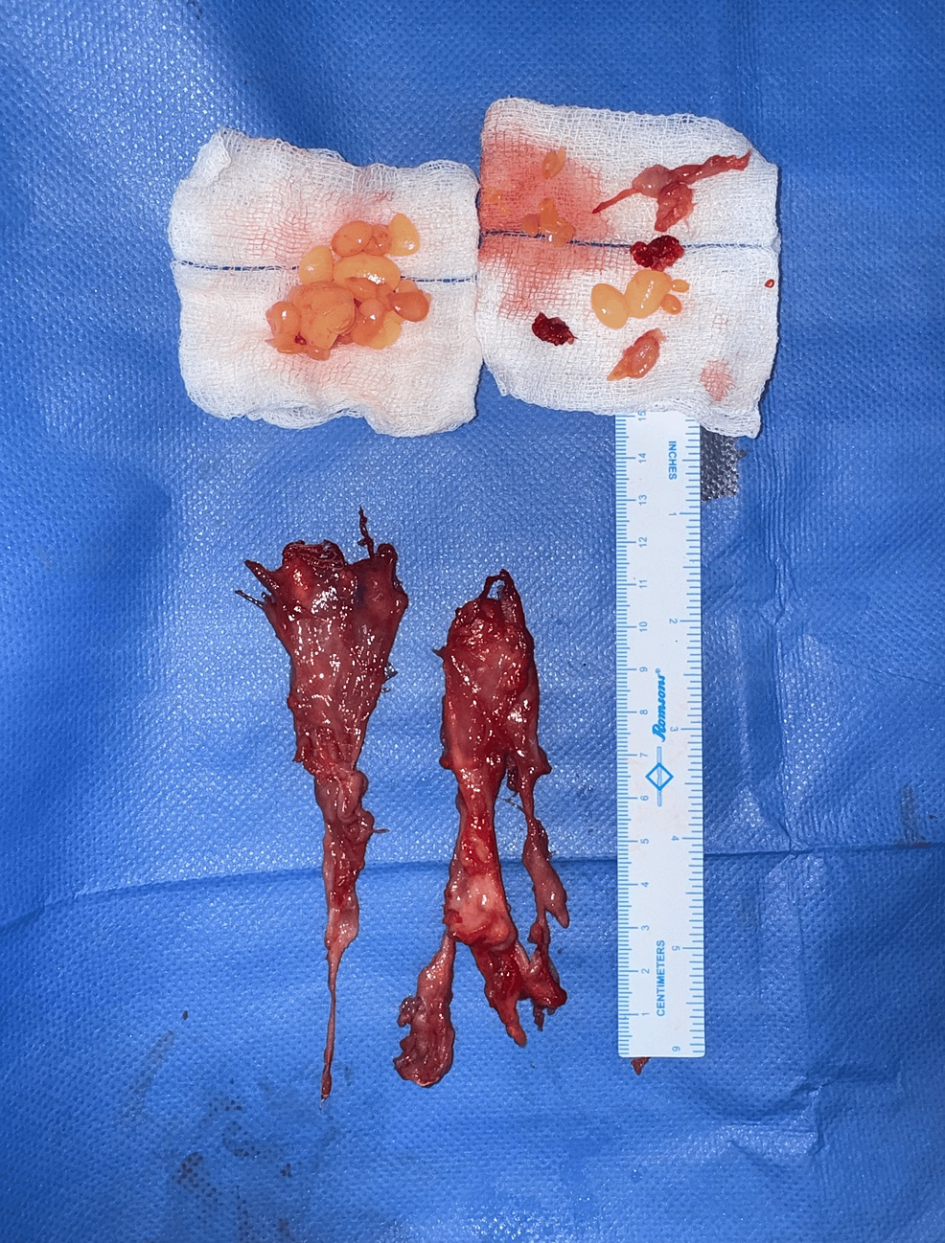
Excised specimen surrounding the synovial sheath

Following inconclusive investigations, a differential diagnosis of CTT, or giant cell tumor of the tendon sheath, was considered due to the evident swelling, ulceration, and nerve compression. Given the need for tissue diagnosis, surgical excision was planned. After obtaining anesthesia clearance, the patient underwent exploration and excision of the soft tissue swelling in the left hand under regional anesthesia. A zigzag incision was made from the tip of the little finger to 2 cm below the thenar eminence (Figure 6). Skin flaps were carefully raised, revealing inflamed tendon sheaths surrounding the flexor digitorum profundus, flexor digitorum superficialis, and synovium. Numerous soft, globular swellings were identified along the tendon sheaths (Figures 7, 8), which were excised meticulously in a piece-meal manner along the length of the tendons. This approach allowed for the careful removal of the swellings while preserving the integrity of the tendons (Figure 9).

**Figure 10:**
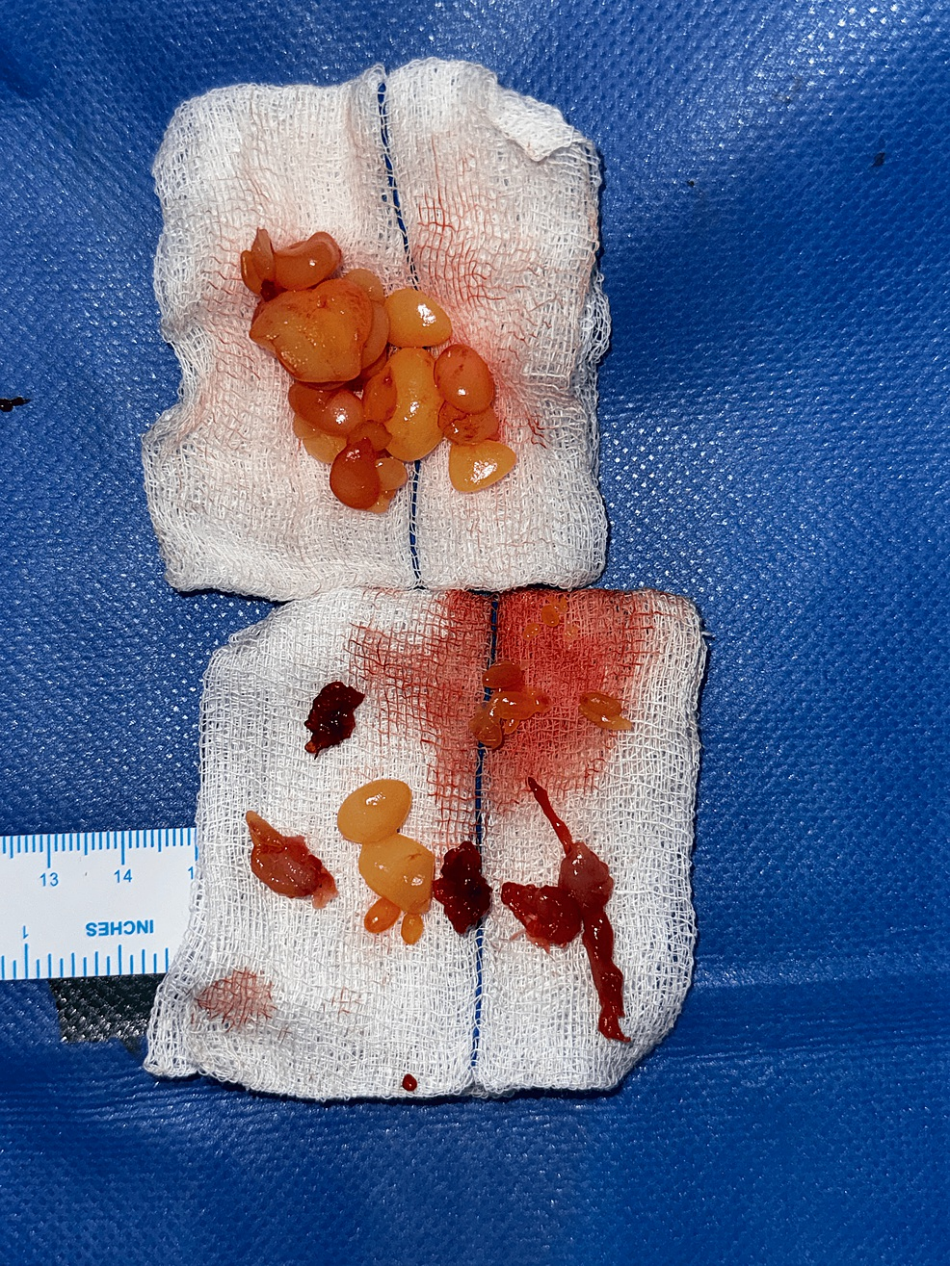
Contents of the swelling consisting of multiple golden-yellow, soft, glistening, and smooth lobules

**Figure 11:**
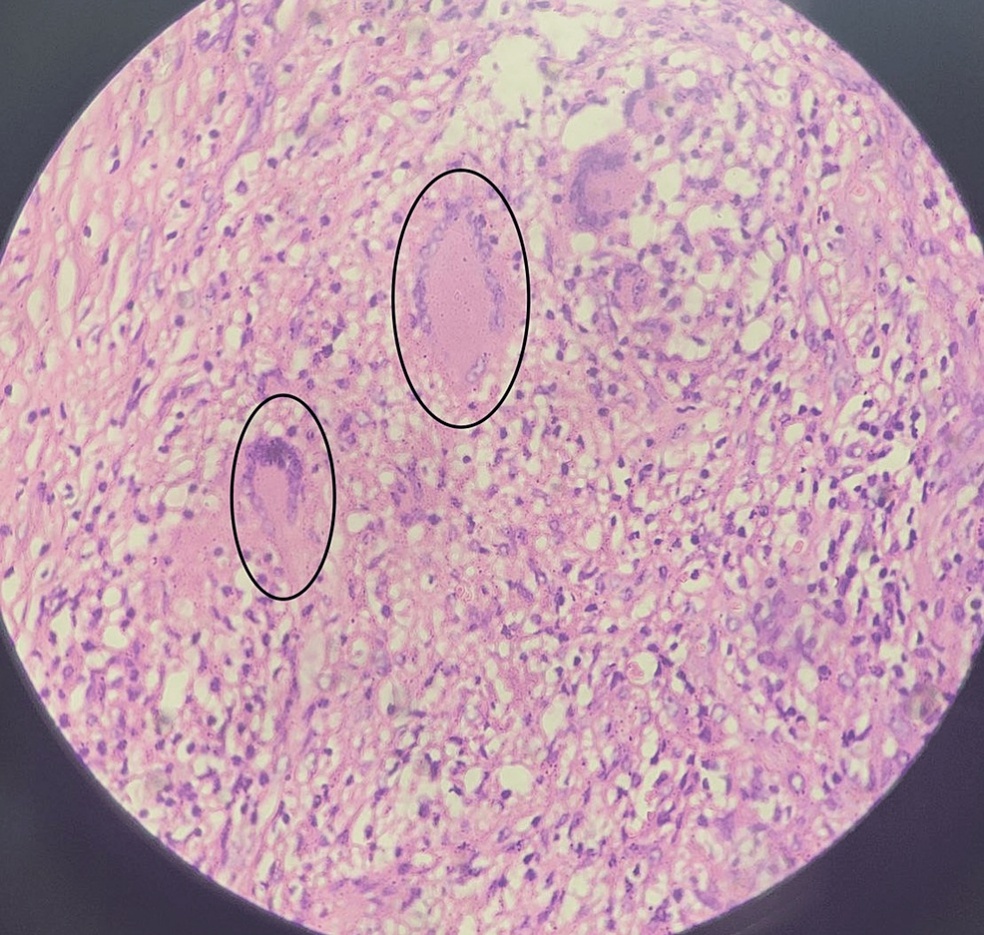
Histopathological image at 10x magnification, stained with H&E, depicting fibrocollagenous tissue with Langhans-type giant cells

**Figure 12:**
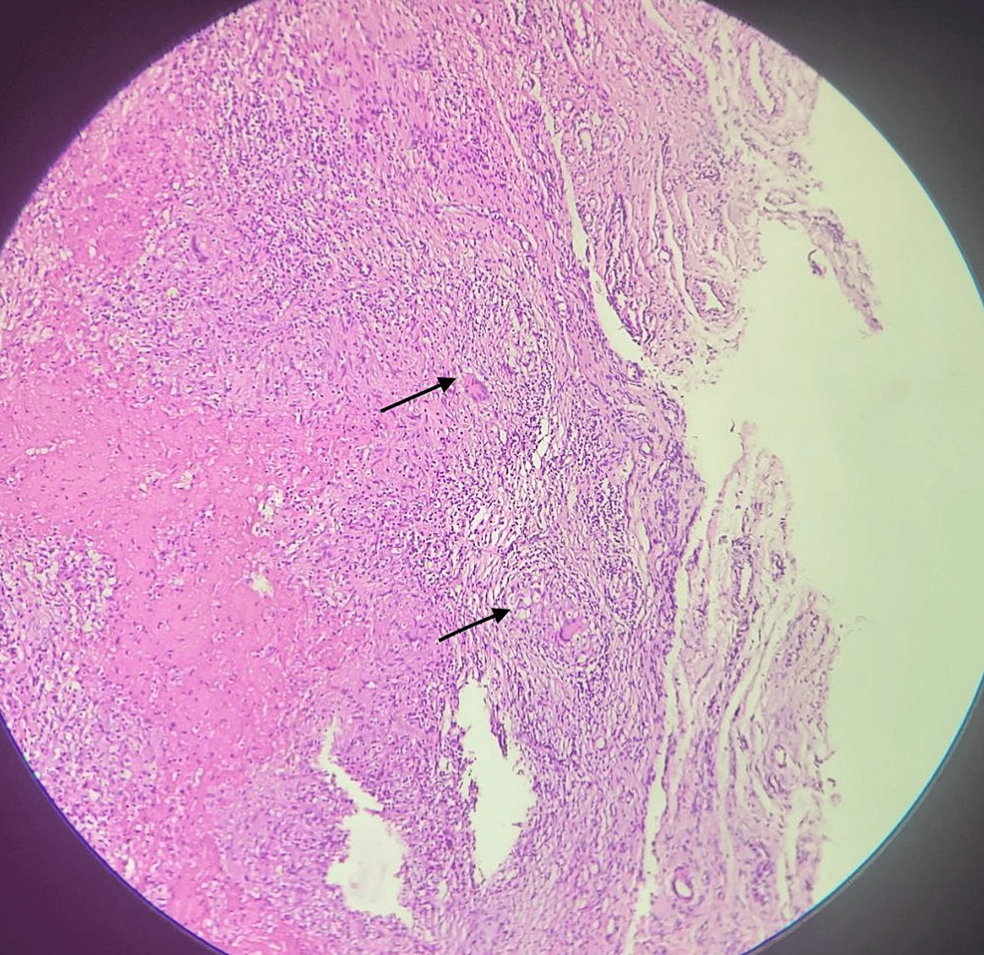
Histopathological image at 40x magnification, stained with H&E, revealing areas of necrosis accompanied by granulomatous inflammation

**Figure 13:**
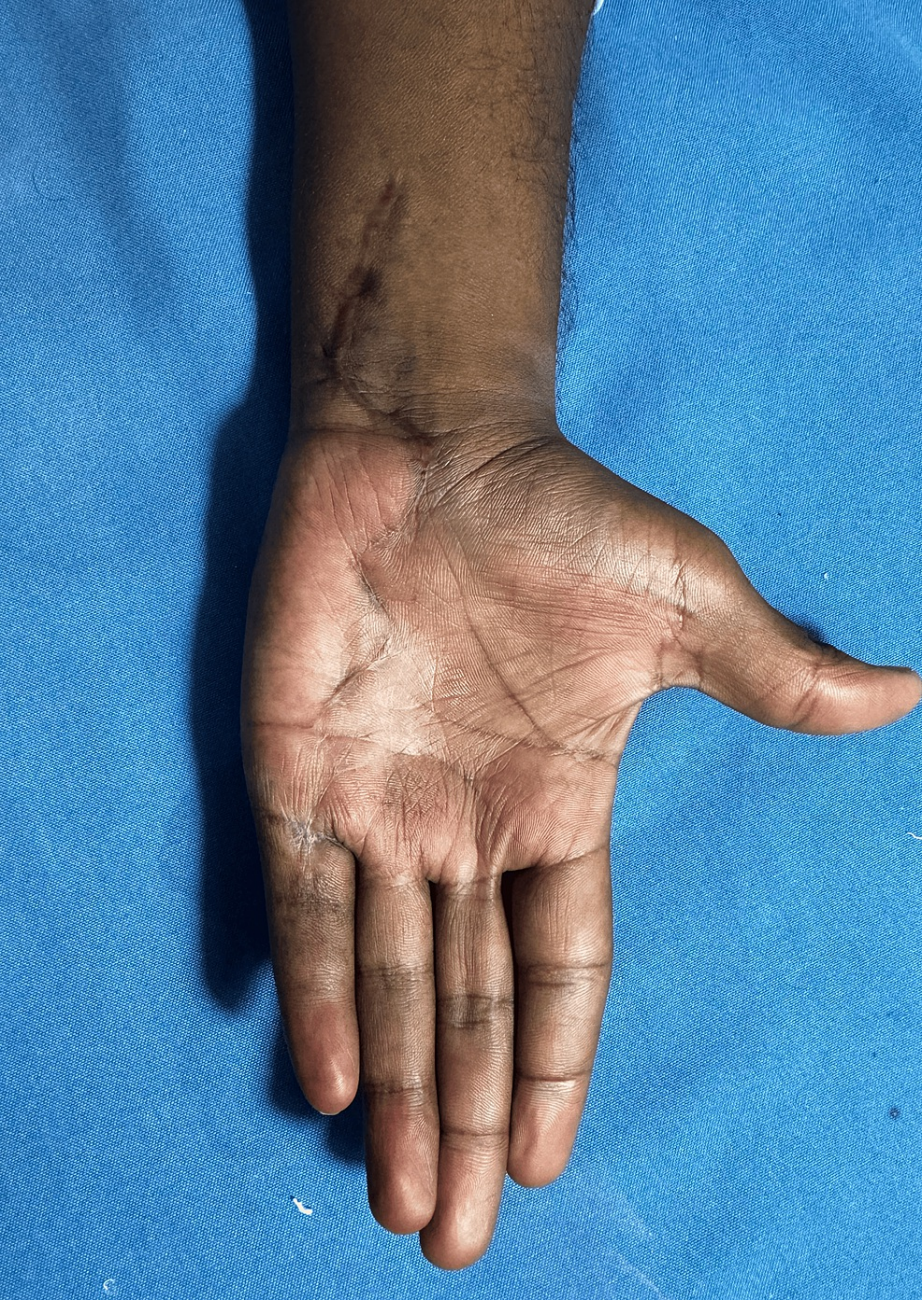
Image of a healthy scar taken three months post-surgery

**Figure 14:**
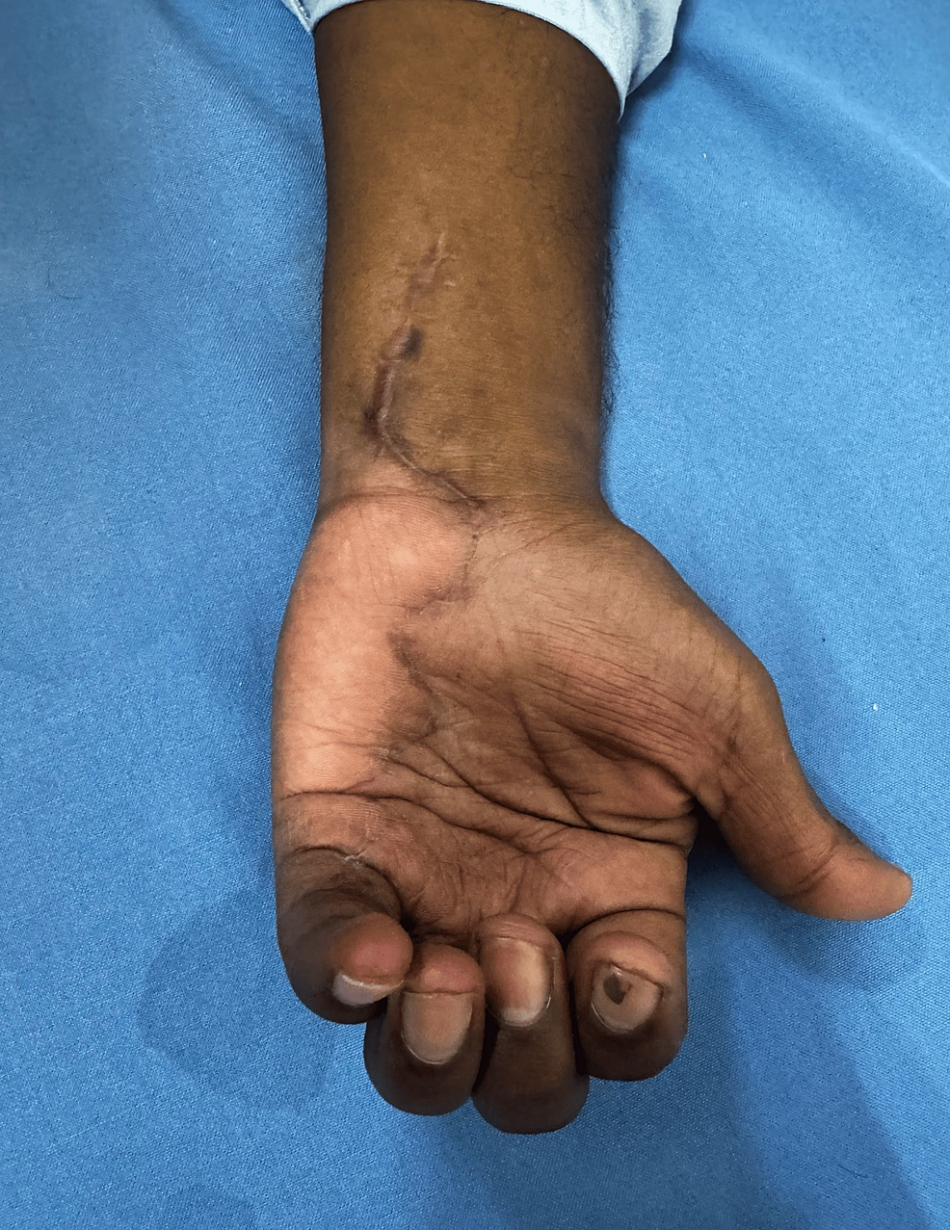
Follow-up image showing the left hand after regaining partial function

The contents of the swelling were multiple soft, glistening, smooth lobules that were golden yellow in color (Figure 10). After a thorough saline wash, hemostasis was secured. Capillary refill time was checked, and the skin was closed with segmuller drains. Specimens of the swelling, along with its contents, were separately sent for microbiology and histopathology. The histopathology report revealed multiple fragments of fibrocollagenous tissue composed of Langhans-type giant cells (Figure 11), along with lymphocytes and a few epithelioid cells, showing areas of necrosis and granulomatous inflammation consistent with Koch’s bacillus (Figure 12). The contents of the swelling displayed eosinophilic material with mostly acellular necrosis, suggestive of rice bodies typical of tuberculous etiology. Postoperatively, his capillary refill time remained normal. He was initiated on an ATT regimen for six months and discharged on postoperative day 4. The patient underwent follow-up visits at one week, one month, and three months post-surgery. The wound had healed with a healthy scar (Figure 13), and he had regained partial range of motion in his left little finger (Figure 14).

## Discussion

In 2022, according to the WHO, approximately 10.6 million cases of tuberculosis are reported annually worldwide, with around 6 million cases affecting males and 3.5 million affecting females [5]. Musculoskeletal tuberculosis has an age distribution ranging from 45 to 60 years [6], although some studies show a bimodal age distribution peaking in the third and sixth decades of life [7]. TS is the disruption of the sheath surrounding the tendon, or synovium. A condition that may be infectious, noninfectious, inflammatory, or idiopathic in nature due to inflammatory pathology can be an autoimmune disease, rheumatoid arthritis, trauma, or idiopathic, in which case it generally affects the wrist. Non-inflammatory TS can be stenosing TS or De Quervain TS. Infectious TS is more common in the flexor tendons than the extensor tendons [8]. The most common causative organism of TS is methicillin-sensitive *S. aureus*, followed by methicillin-resistant *S. aureus *[3], but Mycobacterium tuberculosis is fairly rare [9]. In general, tuberculosis spreads through the lymphatic or hematogenous route [10]. Some patients could have an active tuberculosis infection, whereas others may be asymptomatic and have latent tuberculosis. In both cases, the infection could be primarily pulmonary in origin or could have pulmonary and extrapulmonary manifestations. In extrapulmonary tuberculosis, lymph nodes and pleura are most commonly affected [11]. Although approximately 3% of extrapulmonary tuberculosis involves the bone and joint system, its presentation in the hand is as rare as 1% [12].

Most patients with tuberculous TS present with painless swelling and restricted movements in a particular joint. All labs are generally normal except for raised ESR and CRP. The tuberculin test is positive in some cases, and almost half the patients show normal X-ray findings [13]. Despite the fact that ultrasound identifies the thickening of the synovial sheath, MRI is the most valuable tool to identify nerve compression, bone destruction, and abscesses [14]. In the above case, the obvious swelling and history of nonhealing ulcers led to a diagnosis of tuberculous TS. The clinical findings, along with nerve compression, showed a definite need for tissue diagnosis, and hence surgical excision was planned. Confirmation of tuberculous TS is done by excision, biopsy, and tissue culture. Generally, histopathology shows caseating granulomas with multinucleated giant cells; less than 30% of cases show non-caseating granulomas [15]. Rice bodies may be seen during surgery; however, rice bodies are not characteristic of tuberculous TS. They are also seen in synovial chondromatosis, seronegative arthritis, and rheumatoid arthritis. A single standard treatment route is debatable. Some physicians advise debridement of the surrounding tissue and decompression of the tendon sheath without excision [16]. However, others, like Tuli, recommended four months of ATT and, if unresponsive, to proceed with surgical debridement [17]. Overall, the management is aimed at surgical debridement, followed by ATT with first-line drugs like rifampicin, isoniazid, pyrazinamide, and ethambutol for at least nine months in cases of nerve compression.

## Conclusions

Tuberculous TS of the hand remains an unusual condition and requires good knowledge of its various presentations. Even though it usually involves the flexor tendons, the nonspecific symptoms can delay the proper diagnosis and thus its treatment. Ultrasonography, MRI, and histopathological diagnoses are required to confirm this unusual condition. There is no gold standard treatment for tuberculous TS of the hand; hence, surgical excision and a minimum of six months of first-line ATT are indicated for a good outcome.
